# Effect of condensed tannins from *Leucaena leucocephala* on rumen fermentation, methane production and population of rumen protozoa in heifers fed low-quality forage

**DOI:** 10.5713/ajas.17.0192

**Published:** 2017-11-03

**Authors:** Angel T. Piñeiro-Vázquez, Jorge R. Canul-Solis, Guillermo O. Jiménez-Ferrer, José A. Alayón-Gamboa, Alfonso J. Chay-Canul, Armin J. Ayala-Burgos, Carlos F. Aguilar-Pérez, Juan C. Ku-Vera

**Affiliations:** 1 Facultad de Medicina Veterinaria y Zootecnia, Universidad Autónoma de Yucatán. C.P. 97300 Mérida, Yucatán, México; 2 Instituto Tecnológico de Conkal, Avenida Tecnológico s/n C.P. 97345 Conkal, Yucatán, México; 3 El Colegio de la Frontera Sur (ECOSUR), Ganadería y Ambiente, Carretera Panamericana y Periférico Sur s/n, Barrio María Auxiliadora, San Cristóbal de Las Casas, Chiapas 29290, México; 4 El Colegio de la Frontera Sur, Unidad Campeche, México. Av. Rancho Polígono 2-A, Ciudad Industrial Lerma, C.P.24500, Campeche, México; 5 División Académica de Ciencias Agropecuarias, Universidad Juárez Autónoma de Tabasco, Carretera Villahermosa-Teapa, km 25, R/a. La Huasteca 2a. Sección, C.P. 86280 Villahermosa, Tabasco, México

**Keywords:** Methane, Crossbred Heifers, Volatile Fatty Acids, Forages, Respiration Chambers

## Abstract

**Objective:**

The aim of the experiment was to assess the effect of increasing amounts of *Leucaena leucocephala* forage on dry matter intake (DMI), organic matter intake (OMI), enteric methane production, rumen fermentation pattern and protozoa population in cattle fed *Pennisetum purpureum* and housed in respiration chambers.

**Methods:**

Five crossbred heifers (*Bos taurus*×*Bos indicus*) (BW: 295±6 kg) were fed chopped *P. purpureum* grass and increasing levels of *L. leucocephala* (0%, 20%, 40%, 60%, and 80% of dry matter [DM]) in a 5×5 Latin square design.

**Results:**

The voluntary intake and methane production were measured for 23 h per day in respiration chambers; molar proportions of volatile fatty acids (VFAs) were determined at 6 h postprandial period. Molar concentration of VFAs in rumen liquor were similar (p>0.05) between treatments. However, methane production decreased linearly (p<0.005), recording a maximum reduction of up to ~61% with 80% of DM incorporation of *L. leucocephala* in the ration and no changes (p>0.05) in rumen protozoa population were found.

**Conclusion:**

Inclusion of 80% of *L. leucocephala* in the diet of heifers fed low-quality tropical forages has the capacity to reduce up to 61.3% enteric methane emission without affecting DMI, OMI, and protozoa population in rumen liquor.

## INTRODUCTION

Methane (CH_4_) gas is a byproduct of anaerobic fermentation of carbohydrates in the rumen. It is considered a greenhouse gas with a global warming potential twenty-three times greater than that of CO_2_ [1] and every year 80 million tons are generated from anthropogenic activities. It has been estimated that enteric methane contributes 39.1% of greenhouse gas emissions generated by the livestock sector [2]. Additionally, CH_4_ emissions represent an energy loss which ranges from 2% to 12% of the gross energy consumed by ruminants [3].

In tropical regions, ruminants are usually fed low-quality forages which are characterized by their low concentration of crude protein (CP) and digestible energy, and their high content of neutral detergent fiber (NDF) and lignin, which induces a higher methane convention rate value [4] decreasing the efficiency of energy utilization [5]. Nonetheless, feeding ruminants with legumes favors a lower CH_4_ production [6].

In the tropical regions, there is a large diversity of legume species which contain secon dary metabolites such as condensed tannins (CT), which decreases the protozoa population, methanogenic archaea [7], and the synthesis of enteric CH_4_ [8,9]. *Leucaena leucocephala* (*L. leucocephala*) is one of the main tropical shrub species which contains CT with potential to reduce CH_4_ emissions [10], improve CP digestibility [11], N retention [8,9] and improve the efficiency of energy intake by ruminants [12]. Harrison et al [13] have recently reported higher levels of productivity with cattle fed leucaena in Australia as well as lower enteric CH_4_ emissions to the atmosphere.

The aim of the work hereby described was to assess the effect of supplying increasing concentrations of CT from *L. leucocephala* in the ration on feed intake, molar proportions of volatile fatty acids (VFAs), changes in rumen protozoa population and enteric CH_4_ emissions by heifers fed a basal ration of tropical grass.

## MATERIALS AND METHODS

### Animal care

The animals were treated in accordance with guidelines and regulations for animal experimentation of the Faculty of Veterinary Medicine and Animal Science (FMVZ), University of Yucatan, Merida, Mexico.

### Location

The experiment was carried out at the FMVZ, University of Yucatan in Merida, Mexico. Climate in the region is warm with an average temperature of 26.8°C and average rainfall of 984.4 mm per year [14].

### Experimental animals

Five crossbred (*Bos taurus*×*Bos indicus*) heifers with an average body weight (BW) of 295±6.8 kg were housed in metabolic crates in a roofed building with concrete floor without walls. Before the experiment heifers were internally dewormed with Ivermectin (AGROVET, S.A de C.V. Mexico, Federal District, Mexico) (1%; 1 mL/50 kg BW) and injected intramuscularly with ADE vitamins (1 mL/10 kg BW).

### Experimental design and treatments

A 5×5 Latin square design [15] was used; each period lasted 19 days (13 days for adaptation to management and rations, and six days for measurements of response variables). During the adaptation period (13 days), the animals remained outside the chambers and only during the measurement period (6 days) the intake, digestibility and enteric methane production were measured. Prior to the experiment, animals were adapted to enter the respiration chambers for a period of ~3 weeks to reduce the effect of stress on voluntary intake of dry matter (DM) and behavior in and out of the chambers. Basal ration consisted of fresh forage of Taiwan (*Pennisetum purpureum* [*P. purpureum*]) grass and increasing levels of chopped forage of *L. leucocephala* as a replacement for the *P. purpureum* forage which was homogeneously mixed (Forage-legume) to avoid selection. The chemical composition of the forages is shown in Table 1. Experimental treatments were increasing levels of *L. leucocephala* (0%, 20%, 40%, 60%, and 80% of DM) in the ration. Additionally, heifers were supplemented with a commercial mineral mixture Fogysal Cattle (FOGYSA S.A de C.V. Merida, Yucatan, Mexico) (250 g/heifers/d).

### Response variables

#### Determination of rumen pH and molar proportions of volatile fatty acid

Samples of rumen liquor were taken by an esophageal tube according to Ramos-Morales et al [16]. Rumen liquor was obtained during six days before measurements in each period, six hours postprandial according to recommendations by Hales et al [17] and Bathha et al [18] in order to analyze rumen pH and pattern of VFA’s in the rumen. Samples of rumen liquor were filtered through double layers of cheesecloth to retain large particles. Rumen pH was determined immediately after taking the sample with a portable potentiometer (HANNA Instruments, Woonsocket, RI, USA), previously calibrated with buffers at pH’s 4, 7 and 10. For the determination of molar proportions of VFA’s in rumen liquor, 4 mL samples were taken and added 1 mL of a deproteinizing solution consisting of metaphosphoric and 3-methilvaleric acids. For VFA analysis, the technique proposed by Ryan [19] was employed using a gas chromatograph (Hewlett-Packard, 5890 series III), equipped with a flame ionization detector and a column HP-FFAP measuring 30 m×0.53 mm, temperature of the injector and the detector was in both cases 200°C.

### Measurement of enteric CH_4_

Measurement of CH_4_ was carried out while heifers were housed in respiration chambers [20], with dimensions 2.10 m×1.60 m×3.10 m (height, width and length, respectively) and a total internal volume of 9.38 m^3^. Mass flow generators (Sable Systems International, Las Vegas, NV, USA) pulled out the air from the chamber at a rate of 500 L/min creating a negative pressure of 475 Pa inside the chamber relative to the outside environment.

Measurement of CH _4_ in air samples was carried out with an infrared analyzer (MA-10, Sable Systems International, USA). High purity CH_4_ gas (99.9999% CH_4_; Praxair, Mexico) was released from a small cylinder into the chambers and the recovery rate was assessed to be 100.2% which is comparable to recovery rates obtained in other laboratories [21]. The methane analyzer was zeroed by infusing pure N_2_ and the linearity (r^2^ = 0.9999) of the analyzer response was assessed by infusing increasing concentrations of methane (1,000; 2,500; 5,000; 7,500 ppm) diluted in N_2_ before each run. Heifers were kept inside the respiration chambers at a temperature of 23°C and relative humidity of 55%. Fresh water was available at all times from an automatic drinking bowl and a small fan mixed the air inside the chambers. Methane measurements were carried out during three consecutive days for 23 h continuously during the measurement period of the response variables, inside the respiration chambers intake and digestibility were also measured [20,4]. Data obtained were adjusted to 24 h runs with ExpeData software (Sable Systems International, USA) and converted to liters of methane produced per day.

### Voluntary intake

Heifers were fed *ad libitum* allowing a refusal of at least 15% of the dry matter offered the previous day. Feed refusals were weighed at 0900 h the following day. Voluntary intake was determined by the difference between the amount offered and refused. Feed offered covered an estimated intake of 7.0 kg DM/d.

### Rumen protozoa population

Protozoa in samples of rumen liquor were counted according to the procedures described by Rosales [22]. One mL of rumen liquor was mixed with 1 mL of methylgreen formalin saline solution (35 mL/L formaldehyde, 0.14 m*M* NaCl, 0.92 m*M* methylgreen) and centrifuged at 2,000 rpm for 20 min. Then, an aliquot was taken and introduced to a modified Fusch-Rosenthal chamber (Electron Microscopy Science, Hatfield, PA, USA) (IVD 98/79 CE) and observed under the microscope (Mikon-YS100) (Nikon Instruments Inc, Melville, NY, USA) at 40×. Number of ciliate protozoa was reported as log 10 from total number+1 per mL of rumen liquor. Number of protozoa was estimated with the following formulae [23]: Number of cells per mL^−^^1^ = [(n1+n2+n3+n4+n5)/5]/0.022 mm^3^×10^3^×d. where: n1…n5: number of protozoa per large square and d = dilution factor. Classification of protozoa was carried out according to Ogimoto and Imai [24].

### Chemical analysis

Dry matter was determined in a forced air oven at 55°C for 48 h (constant weight) (#7.007) [25]. Nitrogen (CP = N×6.25) determinations were carried out with a LECCO CN-2000 series 3740 instrument (LECCO, Corporation, Saint Joseph, MI, USA) (#2.057) [25]. Organic matter was assessed by incineration in muffle furnace at 550°C for 6 h (AOAC Method #923.03) and the content of NDF and acid detergent fiber (ADF) were determined using the methods described by Van Soest et al [26]. Concentration of CT in forage samples was performed by the HCL-butanol method described by Makkar [27].

### Statistical analysis

Data on voluntary intake, apparent digestibility, nitrogen balance, protozoa population and CH4 production were subjected to analysis of variance for a 5×5 Latin square design [15] using the procedure PROC ANOVA from SAS (SAS Inst. Inc., Cary, NC, USA) with the model Y_ijk_ = μ+P_i_+A_j_+T_k_+e_ijk_, where Y_ijk_ is the dependent variable, μ is the general mean, P_i_ is the effect of period, A_j_ is the effect of animal, T_k_ is the effect of treatment, and e_ijk_ is the residual error. Statistical differences were declared significant at p≤0.05. Additionally, surface response analysis was carried out to assess the linear, quadratic or cubic effects [28] of the response to treatments (0%, 20%, 40%, 60%, and 80% of *L. leucocephala* in the ration).

## RESULTS

### Voluntary intake

Dry matter intake (DMI, kg/d and g/kg^0.75^) was similar among treatments with different levels of *L. leucocephala* forage, reporting an average of 7.0 (kg/d) and 98.7 (g/kg^0.75^), respectively (p>0.05). Organic matter intake (OMI) followed the same trend as DMI (Table 2). However, crude protein intake (CPI) increased linearly as the level of incorporation of *L. leucocephala* in the ration was augmented (p<0.0001).

### Enteric methane production

The average of methane production in this study was 20.1 L/kg of DMI with the control treatment and as *L. leucocephala* increased in the diet there was a reduced CH_4_ production; with incorporation of 80% of *L. leucocephala* in the ration methane production reduced by 61.6% respect to 0% of legume (linear reduction p = 0.0005). Also, methane emissions expressed as L CH4/d, kg of DM, OM, NDF, ADF intake, kg of digestibility dry matter, and organic matter digestibility (OMD), neutral detergent fiber digestibility (NDFD), an acid detergent fiber digestibility (ADFD) was linearly reduced (p<0.01) as the percentage incorporation of *L. leucocephala* in the ration was increased (Table 3).

### pH, NH_3_-N, and molar proportions of volatile fatty acid in the rumen

No differences were observed in rumen pH as the inclusion of *L. leucocephala* in the ration of cattle was increased (p>0.05). Although rumen NH_3_-N was different between treatments and linearly increased as the level of the foliage of the legume was increased (p<0.01). As from 80% of *L. leucocephala* (36 mg/dL) in the ration, rumen NH_3_-N was significantly increased (138%) with respect to the treatment without incorporation of *L. leucocephala* (15.3 mg/dL) (p<0.05) (Table 4).

Molar proportions of acetic, propionic and butyric acids in rumen liquor were not affected (p>0.05) by the incorporation of *L. leucocephala* in the ration, registering averages of 53.8, 12.9, and 5.7 mmol/L, respectively. When the molar proportion of acetic acid was expressed as percentage, a linear increase (p = 0.037) was detected as the level of incorporation of *L. leucocephala* was increased. Conversely, the molar proportion of propionic acid maintained a linear reduction (p = 0.032) as *L. leucocephala* was increased in the ratio (p<0.0002) (Table 4).

### Rumen protozoa population

Total protozoa numbers, holotrichs, and entodiniomorph in the rumen of cattle were not affected at 6 h after intake of *L. leucocephala* forage (p>0.05) (Table 5).

## DISCUSSION

### Voluntary intake

Intake of tropical foliage that contains CT at moderate concentrations (3% to 6% of DM) improves the nutrient intake of ruminant rations, particularly DM, OM, and CP [29]. In the present work, the incorporation of up to 80% of *L. leucocephala* equivalent to a supply of 16.4 g/kg DMI of CT only had an influence on the greater CP intake (kg/d, CPI/kg digestibility organic matter intake [DOMI]) without affecting DMI, OMI, and acid detergent fiber intake (Table 2). Additionally, reduction in NDF intake might have been influenced by the small amount of NDF contained in the foliage of *L. leucocephala* (580.8 g/kg DM) and as the level of incorporation of tree foliage was increased, the amount of NDF was reduced [6]. These results differ from those of Delgado et al [30] when they included 27% of *L. leucocephala* in sheep rations and observed an increase in the DMI and OMI, similar to results reported by Wahyuni et al [11] who observed an increase in DMI when they included increasing levels of *L. leucocephala*, registering an increase of 30%, when 60% leucaena was incorporated in the ration compared to a control treatment.

It has been pointed out that doses below 2% CT do not af fect DMI [31,32]. Grainger et al [33] found that 1.46% CT in the ration of dairy cows did not affect DMI and Beauchemin et al [32] using 2% CT in cattle rations did not find an effect on nutrient intake. In this study, levels of up to 80% *L. leucocephala* only produces 1.64% of CT per kg DMI in the cattle ration, thus it is unlikely that this level influenced DM and OM intakes.

### Enteric methane production

In this experiment, with heifers fed foliage of *L. leucocephala*, the methane production was lower (11.5 L/kg of DM) [6,34,8] in respect to the heifers without inclusion of *L. leucocephala* (20.1 L/kg of DM) in the ration. In this context, 80% of *L. leucocephala* foliage in the ration, decreased by ~61% the rumen methane production with respect to the control treatment, these results were similar to those obtained [35,8,34].

In the present work, a linear reduction in ruminal methane production was observed as the level of foliage of *L. leucocephala* in the ration was increased (Table 3) and could be associated with CT content. Several studies have demonstrated that inclusion of CT using *L. leucocephala*, reduces ruminal methane production. Tan et al [36] under *in vitro* conditions found a linear reduction in CH_4_ production when they included 20%, 30%, 40%, 50%, and 60% of *L. leucocephala*, reporting a reduction of 63% in CH_4_ production when the level of incorporation of the legume was 60% compared to the control treatment (0% *L. leucocephala*). Rira et al [10] included 75% and 100% of *L. leucocephala* in an *in vitro* system and found reductions of 27.0% and 31.5% in CH_4_ emissions, while Molina-Botero et al [37] incubated 100% of *L. leucocephala* and found a reduction of 31% in CH_4_ emissions when expressed as g/kg DM. Delgado et al [30] included 27% of *L. leucocephala* in sheep rations and observed a reduction of 15.6% in CH_4_ emissions (L/kg DMI).

In the experiment hereby described, a reduction of 39.4% was recorded in CH_4_ production with 40% of DM ration of *L. leucocephala* fed to growing heifers housed in open-circuit respiration chambers. Recent work by Soltan et al [8] included 35% of *L. leucocephala* in sheep rations and found a reduction in CH_4_ production of 14.3% when expressed as L/kg of digestible organic matter, this result is lower than that found in the present experiment with 40% of inclusion of *L. leucocephala* (32.1% less CH_4_). Also, these results differ from results reported by Kennedy and Chamrley [4] who registered a reduction (L CH_4_/kg DMI) in emissions of 13.4% and 20.5% when they included 22% and 44% of *L. leucocephala* in cattle rations.

A higher reduction in methane production per kg of DMI was found in the experiment hereby described with 20% and 40% incorporation of *L. leucocephala* (26.7% and 39.4% less CH_4_/kg DMI, respectively) and when results were expressed as L CH_4_/kg DOMI, our results were similar to the values reported by Kennedy and Chamrley [4] with 22% and 44% of *L. leucocephala* in the ration (19.2% and 28.2% less CH_4_/kg DOMI). However, our results with 80% of inclusion of *L. leucocepha* in the diet differed from that reported by Dias-Moreira et al [34] whoadded 82% of *L. leucocephala* (40 g CT/kg DM) to sheep rations and found a reduction of 26% in rumen methane production. This could be associated with the CT concentrations on the diet. Grainger et al [33] included 1.46% CTs per kg dry matter and found a reduction of 22.3%, and in our present work the inclusion of 1.64% CT per kg of ration DM (80% of *L. leucocephala*), induced a reduction in methane production of 61.3%.

Min et al [31] found that the inclusion of 2% of CT as DM exerted a reduction of 31% in ruminal CH_4_ production. In several experiments using plants containing CTs, similar reductions in CH_4_ emissions to those found in the experiment hereby described have been registered. Puchala et al [35] found that goats fed *Lespedeza cuneata* containing 153 g CT/kg DM reduced enteric CH_4_ emissions by 57.1%. Similar data were reported by Animut et al [38] in goats eating 200, 447, and 613 g DM of *Lespedeza striata* and found a reduction in CH_4_ emissions of 32.8%, 47.3%, and 58.4%, respectively.

This lower digestibility [4,8] could reduce CH _4_ synthesis because the fibrous component on the diet with legume was lower due to their lower amounts of lignified components compared with tropical grasses, so the time of residence in the rumen is shorter [39,40,7]. In this experiment, a reduction in rumen protozoa population was not observed, probably due to the low concentration of CT (<20 g/kg DM; [41]), however, several workers have mentioned that CT can reduce protozoa population but furthermore, they can also reduce the enzymatic activity of protozoa and bacteria [10].

### Rumen pH and molar proportions of volatile fatty acid

Rumen pH was unaffected by the level of inclusion of *L. leucocephala* due to the fact that the forages had a high content of cellulose. The increase in the concentration of rumen NH_3_-N is related to the increase in CP digestibility and it was observed that rumen ammonia concentration in the treatments with inclusion of *L. leucocephala* was above values considered as normal (15 to 20 mg/dL). This is contrary to that reported by Traiyakun et al [42], who included 0%, 25%, 50%, and 75% of *L. leucocephala* in goat rations and found no effect on rumen NH_3_-N concentrations. Osakwe and Steingass [43] observed a reduction in rumen NH_3_-N concentrations as the level of *L. leucocephala* was increased from 0%, 25%, and 50% of ration dry matter, claiming this was due to the reduction in rumen degradation of CP [44]. In the present study, rumen concentrations of acetic, propionic and butyric acids (mmol/L) were unaffected; however, when acetic acid is expressed as a percentage relative to the other VFA, its contribution increased as the level of *L. leucocephala* in the ration was increased. On the contrary, percentage of propionic acid was linearly reduced as *L. leucocephala* in the ration was increased, and the same trend was observed for the proportion of butyric acid. It was observed that the ratio acetic:propionic acid was increased as the level of *L. leucocephala* in the ration was augmented. These results agree with those reported by Tan et al [36] who under *in vitro* conditions incorporated 0%, 20%, 30%, 40%, 50%, and 60% *L. leucocephala*, and observed a linear increase in the concentration of acetic acid, but a reduction in the proportion of propionic acid and an increase in the acetic:propionic ratio in the rumen. Our results are also supported by Tiemann et al [45], who evaluated the molar proportions of VFA under *in vitro* conditions when they mixed *L. leucocephala* with *P. purpureum* and found that *L. leucocephala* resulted in a higher amount of acetic acid but reduced the concentration of propionic acid. Rira et al [10] evaluated *in vitro* inclusions of 25%, 50%, 75%, and 100% *L. leucocephala* and they did not find any effect on the molar proportions (mol/100 mol) of acetic and propionic acids. However, when they included 44% of *L. leucocephala* in Texel sheep rations, a reduction of 5.6% in acetic acid and an increase of 22.8% in the concentration of propionic acid were found. The results of other studies also differ; Soltan et al [44] found that at levels of 50% of *L. leucocephala*, total concentration of VFA was increased by 17.3%, contrary to that found in the present experiment where no effect was observed on any of the main VFA in the rumen. Authors such as Tiemann et al [45] pointed out that the source of CT is an important factor which determines the effect on ruminal fermentation, however, the concentrations, molecular weight and the chemical structure of CTs are all important factors which determine their effect on the molar proportions of VFA [45,46]. It is still possible that the low concentrations of CT (1.64% CT/kg DMI) at the maximum level (80% of ration DM as *L. leucocephala*) of incorporation of the legume, had no effect in the concentrations of VFA when expressed as mmol/L.

### Rumen protozoa population

The precise mechanism whereby CTs reduce CH_4_ production is still uncertain, however CTs have the capacity to reduce protozoa [46] and methanogenic archaea populations [7]. CT could also reduce CH_4_ production by means of indirect mechanisms as the reduction in digestibility of dietary components [42] by forming chemical complexes with carbohydrates and proteins as it was found in this experiment in which a reduction in OMD was recorded as the level of incorporation of *L. leucocephala* was increased as well as by linearly reduced NDFD and ADFD (Table 2).

Several studies indicate that CTs of tropical trees or legumes have the capacity of reducing rumen protozoa [46,47,36,35], and bacteria [7] populations. It is known that protozoa populations are responsible for 37% of CH_4_ emissions [48] and they can degrade up to 50% of the fibrous fraction of feedstuffs [49,50]. Working with protozoa-free animals, Yañez-Ruiz et al [48] found a reduction in CH_4_ emissions. In the experiment hereby described, no effect was detected of CT contained in *L. leucocephala* on rumen populations of holotrich and entodiniomorph, similar data was reported by Sliwinski et al [51] who fed 0.5% and 1.0% CT/kg DM and did not find any effect on these populations in the rumen. These findings are similar to those reported by Rira et al [10] who incorporated 44% of *L. leucocephala* as a source of CT and did not find any effect on rumen protozoa population. However, under *in vitro* conditions, Tan et al [26] included 20%, 30%, 40%, 50%, and 60% of *L. leucocephala* and found a linear reduction in total and ciliate protozoa. Also, Galindo et al [47] found that at inclusions of 30% of *L. leucocephala*, rumen protozoa population was reduced by 39.4%. Puchala et al [35] supplemented goats with *Lespedeza cuneata* (20% CT/kg DM) and observed a reduction of 65% in ciliate protozoa compared to the treatment when only grass was fed. These findings suggest that small doses of CT in *L. leucocephala* (1.64% CT/kg DMI; 80% of *L. leucocephala* in ration DM) have no effect on rumen protozoa population.

## CONCLUSION

As a source of CT, the tropical legume tree *L. leucocephala* has the capacity to reduce enteric methane emissions in cattle without affecting DM and OM intake, while providing a considerably improved intake of rumen fermentable N for the growth of the microbial population. Inclusion of 80% of *L. leucocephala* in the diet of heifers fed low-quality tropical forages has the capacity to reduce up to 61.3% the methane emission without affecting DMI and OMI, protozoa population and the molar concentration of VFA’s.

## Figures and Tables

**Table 1:** Chemical composition of the forages (g/kg DM)

Item	*Pennisetum purpureum*	*Leucaena leucocephala*
Organic matter	930.32	940.5
Crude protein	60.64	160.29
NDF	670.62	580.85
ADF	410.99	420.01
Lignin	70.24	160.0
Secondary metabolites (g/kg DM)		
Total phenols1)	0	7.7
Condensed tannins2)	0	21.0

DM, dry matter; NDF, neutral detergent fiber; ADF, acid detergent fiber.

1) Expressed as g tannic acid eq/kg DM.

2) Expressed as g leucocyanidin eq/kg DM.

**Table 2:** Intake and apparent digestibility in crossbred heifers fed *Pennisetum purpureum* grass and increasing levels of *Leucaena leucocephala*

Items	Percentage of *L. leucocephala* supplemented (% of DM)					SE	Contrast
	
	0	20	40	60	80		L
BW (kg)	293.80	298.80	289.20	298.20	295.40	6.81	
Intake							
DM (kg/d)	7.03	7.15	7.07	7.00	7.00	0.60	ns
OM (kg/d)	6.54	6.70	6.62	6.53	6.50	0.55	ns
CP (kg/d)	0.50	0.70	0.82	0.93	1.22	0.10	**
NDF (kg/d)	4.63	4.50	4.30	4.10	3.83	0.40	ns
ADF (kg/d)	2.85	2.81	2.77	2.67	2.50	0.25	ns
Digestible intake (kg/d)							
DDM	3.90	3.71	3.46	3.46	3.40	0.54	ns
OMD	3.72	3.54	3.32	3.29	3.21	0.52	ns
NDFD	2.60	2.15	1.91	1.77	1.51	0.33	*
ADFD	1.55	1.30	1.12	1.05	0.81	0.22	*

DM, dry matter; SE, standard error; L, linear contrast; BW, body weight; OM, organic matter; CP, crude protein; NDF, neutral detergent fiber; ADF, acid detergent fiber; DDM: digestible dry matter; OMD: digestible organic matter; NDFD: digestible neutral detergent fiber; ADFD: digestible acid detergent fibre.

* p<0.05;

** p<0.01;

ns: non-significant (p>0.05). No significant effects were found for the quadratic and cubic contrasts.

**Table 3:** Methane (CH_4_) production in crossbred heifers fed *Pennisetum purpureum* grass and increasing levels of *Leucaena leucocephala*

Items	Percent of *L. leucocephala* supplemented (% of DM)					SE	Contrast		
	
	0	20	40	60	80		L	Q	C
BW(kg)	293.8	298.8	289.2	298.2	295.4	6.8			
Enteric methane									
L CH_4_/d	137.3	101.2	87.4	74.9	53.5	14.8	**	ns	ns
L CH_4_/kg of DMI	20.1	14.7	12.1	10.5	7.7	2.1	**	ns	ns
L CH_4_/kg of OMI	21.6	15.7	13.0	11.2	8.3	2.3	**	ns	ns
L CH_4_/kg of NDFI	30.4	23.3	20.0	17.9	14.3	3.4	**	ns	ns
L CH_4_/kg of ADFI	49.1	37.2	31.0	27.7	21.9	5.3	**	**	ns
L of methane/kg of digestible fractions intake									
L CH_4_/kg of DDM	37.7	30.3	25.8	22.7	17.3	5.1	**	ns	ns
L CH_4_/kg of OMD	39.6	31.6	26.9	23.9	18.5	5.4	**	ns	ns
L CH_4_/kg of NDFD	56.5	52.4	47.6	48.7	49.2	11.5	ns	ns	ns
L CH_4_/kg of ADFD	92.0	86.1	84.5	88.8	131.5	33.2	ns	ns	ns

DM, dry matter; SE, standard error; L, linear contrast; Q, quadratic contrast; C, cubic contrast; BW, body weight; DMI, dry matter intake; OMI, organic matter intake; NDFI, neutral detergent fiber intake; ADFI, acid detergent fiber intake; DDM, digestible dry matter; OMD, digestible organic matter; NDFD, digestible neutral detergent fiber; ADFD, digestible acid detergent fiber.

* p<0.05;

** p<0.01;

ns, non-significant (p>0.05).

**Table 4:** Effects of CT on ruminal pH, N-NH_3_, and volatile fatty acids (VFA’s) in crossbred heifers fed *Pennisetum purpureum* grass and increasing levels of *Leucaena leucocephala*

Items	Percent of *L. leucocephala* supplemented (% of DM)					SE	Contrast		
	
	0	20	40	60	80		L	Q	C
Rumen pH	6.8	6.9	6.7	6.8	6.8	0.10	ns	ns	ns
Rumen N-NH_3_ (mg/dL)	15.3	22.0	27.3	34.0	36.5	0.07	**	ns	ns
Volatile fatty acids concentration (mmol/L)									
Acetate	52.6	54.8	53.4	52.1	54.4	5.31	ns	ns	ns
Propionate	13.6	13.1	12.8	12.2	12.8	1.14	ns	ns	ns
Butyrate	6.1	6.1	5.6	5.3	5.4	0.47	ns	ns	ns
Isobutyric	0.4	0.36	0.4	0.37	0.3	0.04	ns	ns	ns
Valeric	0.3	0.34	0.4	0.4	0.4	0.04	*	ns	ns
Isovaleric	0.2	0.2	0.2	0.2	0.2	0.04	ns	ns	ns
TotalVFA	73.5	75.0	72.9	70.7	73.8	6.90	ns	ns	ns
A:P	3.8	4.1	4.1	4.2	4.2	0.10	*	ns	ns
Molar proportions of VFA (%)									
Acetate	71.4	72.9	73.1	73.5	73.5	0.67	*	ns	ns
Propionate	18.6	17.6	17.7	17.4	17.5	0.33	*	ns	ns
Butyrate	8.5	8.3	7.7	7.6	7.5	0.41	ns	ns	ns
Isobutyric	0.5	0.5	0.5	0.5	0.5	0.05	ns	ns	ns
Valeric	0.4	0.4	0.5	0.6	0.6	0.04	**	ns	ns
Isovaleric	0.3	0.3	0.3	0.3	0.3	0.06	*	ns	ns

CT, condensed tannins; NH_3_, ammonia; DM, dry matter; SE, standard error; L, linear contrast; Q, quadratic contrast; C, cubic contrast; A:P, acetic:propionic relationship.

* p<0.05;

** p<0.01;

ns, non-significant (p>0.05).

**Table 5:** Population of rumen protozoa (log_10_ cells/mL) in crossbred heifers fed *Pennisetum purpureum* grass and increasing levels of *Leucaena leucocephala*

Items	Percent of L. leucocephala supplemented (% of DM)					SE	Contrast		
	
	0	20	40	60	80		L	Q	C
Population of rumen protozoa									
Holotrich (log_10_)	4.0	4.1	4.1	3.9	4.1	0.18	ns	ns	ns
Entodiniomorph (log_10_)	5.5	5.3	5.5	5.5	5.5	0.07	ns	ns	ns
Total protozoa (log_10_)	9.5	9.4	9.6	9.3	9.6	0.19	ns	ns	ns

DM, dry matter; SE, standard error; L, linear contrast; Q, quadratic contrast; C, cubic contrast.

* p<0.05;

** p<0.01;

ns, non-significant (p>0.05).
